# Nanotechnology in Dental Sciences: Moving towards a Finer Way of Doing Dentistry

**DOI:** 10.3390/ma3031674

**Published:** 2010-03-08

**Authors:** Vuk Uskoković, Luiz Eduardo Bertassoni

**Affiliations:** 1 Division of Biomaterials and Bioengineering, Department of Preventive and Restorative Dental Sciences, School of Dentistry, University of California, San Francisco, CA, USA; 2 Biomaterials Research Unit, Faculty of Dentistry, University of Sydney, Sydney, Australia; E-Mail: lbra5373@uni.sydney.edu.au (L.E.B.)

**Keywords:** dentistry, nanomaterials, nanoscience, nanotechnology

## Abstract

Nanotechnologies are predicted to revolutionize: (a) the control over materials properties at ultrafine scales; and (b) the sensitivity of tools and devices applied in various scientific and technological fields. In this short review, we argue that dentistry will be no exception to this trend. Here, we present a dynamic view of dental tissues, an adoption of which may lead to finer, more effective and minimally invasive reparation approaches. By doing so, we aim at providing insights into some of the breakthroughs relevant to understanding the genesis of dental tissues at the nanostructural level or generating dental materials with nanoscale critical boundaries. The lineage of the progress of dental science, including the projected path along the presumed nanotechnological direction of research and clinical application is mentioned too. We conclude by claiming that dentistry should follow the trend of probing matter at nanoscale that currently dominates both materials and biological sciences in order to improve on the research strategies and clinical techniques that have traditionally rested on mechanistic assumptions.

## 1. Introduction

The reason for the omnipresence of the word “nano” as one of the most attractive prefixes in the contemporary materials science is simpler than it seems [1,2]. Namely, the progress of humanity is underlain by a continual increase in sensitivity of human interactions with their physical surrounding. As the human societies evolved, the critical length of cutting-edge functional devices has shifted from millimeter to micrometer to nanometer scale. With the scientific ability to control physical processes at nanometer scale, we have entered the era of research and application of nanoscale phenomena. Finally, as material properties often significantly alter following the micro-to-nano shift in the scale at which critical boundaries are found, a new field was born to explain these rather strange phenomena, named nanoscience; the application of its discoveries is known as nanotechnology.

**Figure 1:**
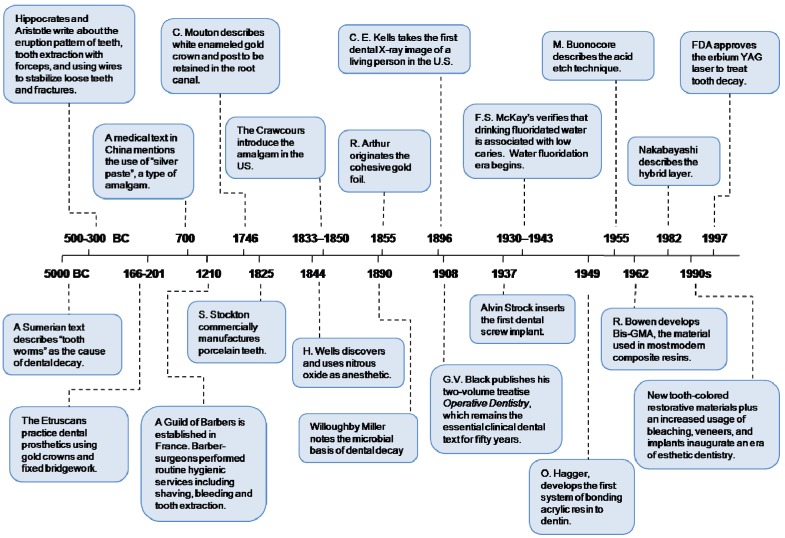
Dental Sciences, the shift from macro to nano (modified from http://www.ada.org/ada/about/history/ada_timeline.asp).

Many fields of science have throughout history rapidly made advantage of tools and techniques that allowed for the design of material properties at a finer scale. Many are hopes that nanotechnology will likewise bring tangible benefits to dentistry, from the bench to the clinical level [3]. As described by Saunders [3], the subject of comparing anticipated *versus* realized in the transition of an emerging technology to the actual practice is not new; however, the pace of application of nanotechnology to dentistry has been less than revolutionary. In Figure 1 we present a timeline showing some of the significant advances in dentistry that illuminated the road for the shift from ‘macro’ to ‘nano’ in dental sciences. It is noticeable that increases in the versatility of scientific knowledge and the ability to control physical processes at a finer resolution naturally led to more information and, henceforth, to more questions. The broader our knowledge, the more amazement arises in face of the natural wonders [4,5]. The same could certainly be said for the field of dentistry. The historic progress in this area naturally goes hand-in-hand with many new questions and challenges that provide opportunities for improvement.

Figure1 illustrates the comparatively moderate progressiveness of dentistry throughout the history. This progress, admittedly, has been slower than might be considered desirable for those who would wish to put a cutting-edge technology to clinical use. For example, early descriptions of the extraction of teeth with the use of forceps by Hippocrates and Aristotle date back to 500–300 BC, a technique that has remained essentially unchanged up to this date. Likewise, restorations with amalgam and gold date back to years 700 and 1746, respectively, and are still a part of our clinical setting without much change.

In this review, we argue in favor of the fact that the upcoming methodologies in dental sciences are no exception to the trend of focusing onto ever finer details in material structures studied in parallel with maintaining the line of progress. In such a way, many routinely used approaches, reparative and exploratory alike, which have traditionally been used in practice will be substituted with finer, more precise and sensitive methodologies. Important questions can be raised about the true benefits of the firm reliance on research strategies and clinical techniques based on the traditional restorative mechanistic assumptions that currently dictate the dental sciences. The aim of this paper is to subject these assumptions to scrutiny and offer a few guidelines that fall into the scope of contemporary nanoscience and nanotechnologies as a way of improving and transforming them into solid bases for novel methods in dentistry.

## 2. The Impact of Nanotechnologies

Nanotechnologies are on the verge of initiating extraordinary advances in biological and biomedical sciences. These would be associated with both providing the tools for improved understanding of fundamental building blocks of materials and tissues at the nanoscale and designing technologies for probing, analysing and reconstructing them. It is not surprising that the development of novel technologies provides the foundations for creation and application of newer and more advanced ones. Expansion of novel technologies, particularly those involved in enriching methods of research, have already changed the way we view and define the standards of high-quality dental materials, tools and practices. A particularly interesting example comes from the improvement of our understanding of micro- and nano- leakages in resin-based restorations, resulting from the development of research methods that allow for visualization of material structures at nanometer scale resolution [6]. Nowadays, they allow us to bring into question the true benefits of our obstinate reliance on the otherwise untouchable concepts. Yet another, more recent example brings into mind the newly proposed technology to evaluate the quality of collagen-based mineralized tissues, such as bone and dentin [7,8,9]. Namely, the quality of mineralized tissues was traditionally evaluated on the basis of the amount of the mineral present in the tissue. The latter could be easily measured using either the clinical X-ray or lab-based mineral density probing techniques. However, recent studies have proven that the functionality of mineralized tissues (which is partly—and perhaps more importantly—biomechanical) is rather dependent on the location and interaction of the mineral with the organic components of the matrix, and not the mineral content *per se* [7,8,9]. It is only through the structural synergy of the mineral and organic components of hard tissues that hardness and stiffness of an inorganic, ceramic material combined with toughness of an organic solid may transcend brittleness of the former and softness of the latter, such as what we see in bone or dentin. These observations represent the need to shift the concepts of diagnosis, treatment and prognosis of dental caries and remineralization of diseased dental tissues towards more progressive ones, which would embrace novel techniques and research strategies (such as already adopted ones [10]), as well as more effective clinical treatments.

It is interesting to recognize at this stage how continual evaluation of clinical concepts becomes allied to the most recent advances in basic research. Perhaps, the connection between basic research and clinical practice is what some dental practitioners fail to recognize. And yet, clinical environment has many times been evidenced as fruitful and essential for the proper evolution along the line of basic research in life sciences. Feedback that researchers receive from clinicians and *vice versa* is essential for the advancement of both fields. Basic research is the first step that leads to a better patient care, which is the final step of any biomedical endeavors. On the other hand, a timely feedback about the pros and cons of materials and technologies in usage from a clinical perspective is critical in directing the research efforts along the right path. As pointed out by Charles Bertolami, “Is the calling to be an outstanding clinician really any different from the calling to be an outstanding scientist? The passion to know is common to both. In the case of the clinician, that need serves the interest of the individual patient. For the biomedical scientist, that need serves the interest of all patients” [11]. Neglecting the need to engage students in the first-class research on the account of extending the time spent in clinic leads many to opine how “too many dental schools are on roller coaster speeding towards majestic mediocrity” [12]. With such an approach, a vicious circle is created in which placing emphasis onto clinic income, dental students and faculty spend more and more time in the clinics than in front of the lab bench, thereby withdrawing their interests from the intensive research. Reforms of the dental education are, therefore, suggested as the key to opening scientific breakthroughs a more immediate path to the regular dental practice [13,14]. It is, on the other hand, valid to point out that nanotechnologies slowly make their way from the lab bench to any other technological or medical field. This is hampered not only by slow progress in understanding the basics in control of nanoscale phenomena, but by strict regulations in the translational stages too [15,16,17]. This, however, offers a controlled environment for the timely identification of weaknesses and strengths, which are all critical when it comes to introducing a new material or technology into the clinical setting.

As we see, nanotechnologies have favoured our understanding of dental tissues at the nanoscale and enabled the design of materials with ultrafine architecture. There is a prospect that probing the structure of dental tissues at ever finer size scales and using the dynamic resolution capabilities of advanced nano tools will give us answers to some of the puzzles that occupy dental researchers of the day. In the next section, we will point out some of the significant advances in dental research that revealed the nano perspective of dental tissues. When viewed at the nanoscale resolution, these structures, otherwise considered as static and stagnant, were seen as exposed to fascinating structural dynamism. Moreover, we will try to yield insights into how this adoption of nanotechnologies may guide dentistry, particularly the reparative aspects of it, to finer, more effective and minimally invasive approaches. So let us start off by describing the cases of dental hard tissues that traditionally used to be viewed as structurally stagnant, but when observed at the nanoscale spatial resolution gained a markedly more dynamical physical appearance.

## 3. Dynamic View of Dental Tissues

### 3.1. The case of enamel

Enamel is composed of 92–94 vol % fibrous apatite crystals with approximately 20 nm in diameter and ultrahigh length-to-width aspect ratios [18]. Precise spatial arrangement of these fibers gives rise to a superstructural organization with symmetry displayed at various size scales, ranging from nano to micro [19]. Morphogenesis of enamel proceeds through interaction between nanospheres or nanofibers of amelogenin, the main protein of the developing enamel matrix, and the growing crystals of apatite [20]. Understanding the principles that govern the self-assembly of amelogenin at the nanoscale thus proves to be essential for understanding the genesis of this complex tissue [21,22]. High resolution electron microscopies and other *in situ* spectroscopic and light scattering techniques [23,24,25], which probe the structure of the protein and mineral components of the developing enamel matrix at the nano level, are thus increasingly employed to understand the mechanism of amelogenesis and possibly replicate this process *in vitro* [26]. With the advancement of real-time nanoscale visualization techniques [27,28], there is a prospect that a clearer picture of these highly dynamic phenomena that govern the formation of dental and other biological tissues will be formed.

Many questions regarding the fine structural details and their functional role in enamel exist. The exceptional roughness of enamel crystals and biological apatite in general, comprising surface irregularities of the order of size of single unit cells is another one of these. Hypothetically, their role is to increase the protein binding in the process of biomineralization [29]. The same can be said for the series of discrete and alternating domains of variously charged (in both magnitude and sign) points along the surfaces of enamel crystals. Then, water, lipids and various peptides account for the non-mineral 2–4 wt % of enamel composition. Although the purpose of this organic matter is usually ignored, it has been suggested to have a functional role in terms of improving the strength of enamel as a whole [30]. As a comparison, crystalline matter in the spine of a sea urchin contains only 0.02 wt % of glycoprotein (~10 proteins per 10^6^ unit cells), but this tiny amount of organic additive remarkably enhances the resistance of the material to fracture [31]. It also modifies the fracture mechanism, making the spine break conchodially like a piece of glass rather than along the low-energy cleavage planes [32]. The fact that some components of the mature enamel, such as enamelin and tuftelin, are full proteins that “survived” the proteolytic digestion during amelogenesis can be taken as a sign of their functional role [18]. Despite being three times less hard than geological apatite, enamel is three times tougher, and that may be not only due to its intrinsic crystalline order, but due to the influence of organic components as well. Entrapment of such a small concentration of macromolecules can increase toughness in an otherwise brittle ceramic material, presumably through absorption of energy from the propagating cracks and deviation of their path. This is why some research groups are beginning to treat enamel as a composite ceramic material despite its low content of organic matter [33].

This perspective goes against the grain of the classical assumption that due to its highly mineralized composition, enamel presents the only tissue in the vertebrate body that, once formed, does not depend anymore on the biological supply of nutrients. All other tissues are built in such a way that every single cell within them needs to be no further than 200 μm away from a blood vessel [34,35]. Enamel is a selectively permeable membrane that allows water and certain ions to pass via diffusion or osmosis. Thus, the presumed non-reparative character of enamel could be reasonably brought into question. In view of this, it becomes apparent that ultrafine precipitation techniques ought to present the subjects of main interest of the contemporary dental research. Cavities start appearing on a small scale, and it is at that point that they should ideally be tackled, as the decay process at a small scale can be made reversible without using aggressive, excavation procedures. Tooth damages at various scales normally require different biomaterials for their reparation, but from the remineralization perspective, the line between preventive and restorative approaches might possibly be erased. Such techniques have a much higher prospect of yielding not only temporary solutions that render the patients treatment-dependent, but the ones that may offer restoration of teeth to their natural forms. Fostering understanding of the dynamical nature of enamel should thus give rise to finer reparative techniques. For instance, understanding what molecules are able to pass through the biological barrier that enamel offers against the oral environment, as first investigated by Dibdin [36], might offer insights into drug delivery techniques that can give guidance in reaching more advanced approaches for enamel tissue engineering and regeneration.

### 3.2. The case of dentin

**Figure 2:**
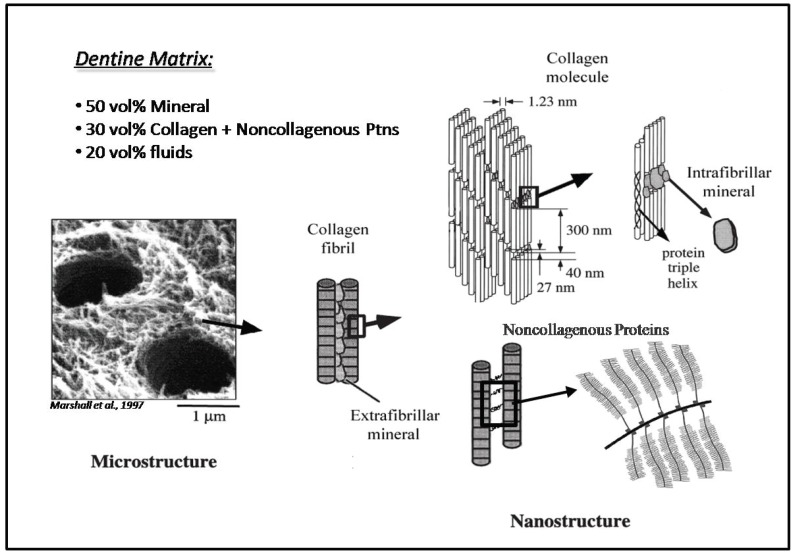
From left to right: SEM image of a fixed, demineralized dentin matrix showing the collagen fibrils. In the schematic on the left, collagen fibrils show the extrafibrillar mineral between fibrils. In the next schematic to the right, the collagen molecules show the 40 nm gap zones and 27 nm overlap zones resulting in the typical 67 nm periodicity of a collagen fibril. The length of the collagen protein triple helix is 300 nm. On the upper right, the intrafibrillar mineral is represented sitting in the gap region between the collagen molecules. The lower middle schematic shows noncollagenous proteins linking collagen fibrils and isolated on the far right. Figure not drawn to scale. Modified from Bertassoni *et al.* [7].

The microstructure of dentin, a composite mineralized tissue, suggests the necessity of a hierarchical approach to the understanding of its mechanical properties [10,37]. The dentin matrix (Figure 2) is mainly composed of type I collagen fibrils with associated noncollagenous proteins, forming a three-dimensional organic scaffold that is reinforced by mineral. The mineral is a nanocrystalline hydroxyapatite that is partitioned according to its location with respect to the collagen fibrils into: extrafibrillar mineral, which is located in the spaces separating the collagen fibrils [38,39,40], and intrafibrillar mineral, which is mainly in the gap regions of the fibrils extending between tropocollagen molecules [41,42,43]. There is uncertainty over the specific morphology of the mineral crystallites. Kinney *et al.* [44] performed a small-angle X-ray scattering analysis on the apatite crystallites in dentin and suggested that the mineral particles are of rod-like shapes near the pulp and are more plate-like shaped, with approximately 5 nm in thickness, near the dentin-enamel junction. Similarly, transmission electron microcoscopy (TEM) investigations [45] confirmed early observations by Boyde [46], indicating the presence of needle-like crystallites in the intertubular dentin region. On the other hand, Lowenstam and Weiner [47], also using TEM, evaluated the ultrastructure of crystallites in bone (which is associated with a similar model of mineralization) after the removal of its organic structures and suggested that the average length and width of the crystallites are 50 and 25 nm, respectively, with an approximate thickness of approximately 2 nm, resembling plate-like structures. It is noteworthy that the current concepts of restorative dentistry ignore most of the recent findings that elucidate the structure and function of dentin at a nanometer scale, which suggests that the modernity of the technologies currently used may be brought into question.

### 3.3. What are we really bonding to?

The concept of hybrid layer formation, first proposed by Nakabayashi *et al.* in 1982 [48], has long been considered responsible for the success of dentin bonding. Hybrid layers form when adhesive co-monomers infiltrate demineralized (*i.e.*, acid-etched) dentin collagen fibrils. However, many studies have shown that nano-leakages, which are only around 20–100 nm wide [49,50] compared to 10–20 μm wide micro-leakage gaps, occur within the hybrid layer even in the absence of gap formation. Although the spaces are too small to allow for bacterial penetration, they are large enough for enzymes and water to enter, which has been suggested to be the pathway for degradation of resin/dentin bonds over time, decreasing dramatically the long term prospects of resin-based restorations. The original interpretation of nano-leakage was that the silver dye used for the leakage studies occupied nanometer-sized spaces around naked collagen fibrils, where resin failed to infiltrate, or where residual water had not been displaced by the adhesive resin [51]. Later TEM work demonstrated that water can pass from dentin, around resin tags, to form water-filled channels that extend from the hybrid layer and into the overlying adhesive [52,53], suggesting that they might act as potential sites for hydrolytic degradation of resin/dentin bonds. On the other hand, it has been shown that the tensile bond strength (TBS) to dentin evaluated using an alcohol-based dentin self-priming adhesive was significantly higher for wet dentin than for the dry one [54], confirming the earlier observations that implied a role that water plays in ensuring the healthy mechanical properties of both dentin [55] and bone [56]. Hydrated dentin is, for example, shown to dramatically degrade in toughness following its dehydration [57]. Significant enthalpic contributions to the thermal stability of non-globular proteins, including collagen, come from the water molecules that form an aqueous scaffold around the surface of the triple helix [58]. One of the essential properties of such fibrous proteins is a relatively high sensitivity to solvation effects compared to the globular ones, which is a natural consequence of a larger number of amino acids exposed on the surface and in contact with the surrounding solvent. The collapse of the collagen scaffold following its dehydration has thus been suggested as the cause for impeded penetration of bonding resins [59]. An improved understanding of the nanostructure of collagen fibrils in dentin would lead to re-interpretation of nano-leakage occurrence and may provide insights into the fundamental limitations of adhesive dentistry. Again, in the original concept of hybrid layer [48], the co-monomers (HEMA, TEGMA, and occasionally UDMA) infiltrate demineralized dentin collagen fibrils. With TEGMA as an example, we may calculate the approximate length of a single extended monomer molecule based on the distance between the atomic bonds (C-C ≈ 120 pm, H-C ≈ 106 pm, and C-O ≈ 143 pm) that form its structure, and realize that the approximate value is 2.2 nm per monomer molecule. Lees (1987) [53] has shown that the lateral spacing of the tropocollagen molecules that form collagen fibrils (Figure2) range from ~1.55 nm when wet and ~1.1 nm when dry. In light of these observations, one may conclude that the intrafibrillar spaces of collagen fibrils would hardly be fully occupied with adhesive monomers, and would, therefore, be filled with water and possibly proteolytic enzymes. Therefore, even when the bonding technique is applied efficiently and used as recommended, the material might not be effective in filling the entire collagen structure, thus becoming compliant to hydrolysis.

## 4. “Small Is Beautiful” of Dental Science: Small Structures, Great Strength

As the ancient alchemists would have reminded us, the whole Cosmos is reflected in the tiny parts thereof and *vice versa* [60]. Focusing our research attention on small details of the physical reality, we are often not aware of the breadth of the potentialities dormant in them. For example, owing to its more periodic structure, significantly simpler than bone, as well as to a comparatively high accessibility, dentin is often used as a model for studying mineralization in bone at the finest, atomic and molecular scales [61,62,63]. Due to the similarity in composition between dentin and bone, any novel insights in the mineral/protein interactions that govern the structure and functionality of dentin may be applicable in reparation of hard tissues in general. Studies of amelogenesis and geneses of other dental tissues aid in fundamentally understanding protein-mineral interactions *per se* [64], which are increasingly used for the synthesis of advanced functional ceramic [65], as exemplified by the synthesis of a few ceramic materials, previously obtained only through high-temperature annealing, by precipitation at room temperature in the presence of short peptides derived from phage display libraries [66,67].

Yet, each detail of the oral microflora abounds with multiple molecular species that define the susceptibility of the subject to disease [68]. Despite that, large varieties in preservation and toughness of teeth among different individuals could not be explained in terms of genetic predispositions only. A synergy of environmental factors and internal immunological traits defines the health of a given organism. Needless to add, complexity of the interaction between soft and hard tissues in this sense reveals itself as particularly important since proper synergy of numerous macromolecular and cellular species are responsible for preservation of healthy oral cavity [69].

Despite centuries of work, dating back to Galileo, the molecular basis of toughness and strength of hard tissues for long remained largely a mystery [70]. A great deal was known about bone, dentin and enamel microstructures and the microcracks that are precursors to their fracture, but little was known about the basic mechanism for dissipating the energy of an impact to keep tissues from fracturing. It has now become increasingly clear that the biomechanics of mineralizing tissues not only depends on the role of the mineral phase in providing stiffness, but it is also related to the interaction between its organic structures. As already pointed out, organic components of bone [70,71], enamel [30] and dentin [72] have been shown to dissipate large amounts of energy under stress. In general, these organic structures work as a ‘glue-like’ material either between mineral crystallites or collagen fibrils in the matrix. Such ‘glue-like’ components are mainly proteins that may deform by gradual unfolding of their domain structures, thus absorbing energy before the peptide backbone is directly stretched [30]. An interesting perspective on these findings is that structures that present the lowest volume fraction in enamel, dentin and bone and also represent the smallest components of the tissue matrices are perhaps the most important in guaranteeing their highest durability. These recent findings may dramatically change our understanding of diseased teeth, since no information exists with regards to changes that occur with these nanostructures in teeth attributed with a hindered biomechanical functionality, such as in dental caries, aged teeth and teeth with a compromised mineralization (fluorosis, dentinogenesis imperfecta, hypoplasia, *etc.*).

As shown in Figure 1, nanotechnologies started to be efficiently applied in dentistry in the early 1970s with the beginning of the era of microfills. Microfilled composites comprise silicon dioxide filler particles with less than 100 nm in diameter in conjunction with prepolymerized organic fillers, aggregated by crushing them into larger filler particles. Nowadays, the most commonly used resin composites, *i.e.*, microhybrids and nanofilled composites, comprise filler particles ranging from approximately 20 to 600 nm. Other dental nanotechnologies rely on the delivery of molecules that facilitate tooth structure remineralization by means of non-invasive dental techniques that forestall caries, the latter being an active area of nano-research in dentistry. Much of this work involves nanoparticles in conjunction with resin-based composite (RBC) systems. Xu *et al.* reported [73] RBCs containing calcium fluoride nanoparticles in a whisker-reinforced resin matrix with sustained fluoride release values exceeding those of conventional and resin-modified glass ionomers. In a different direction, casein phosphopeptide amorphous calcium phosphate (CPP-ACP) are nanoparticles that bind to biofilms, plaque, bacteria, hydroxyapatite and the surrounding soft tissue, localizing bio-available calcium and phosphate and serving as mineral precursors for remineralization [74]. In agreement with Saunders [3], we too suggest that the most tempting venue for speculation on the next phase of nanorestoration of tooth structure is that of nanotechnology mimicking processes that occur in nature, known as biomimetics [75,76].

Quantum dots offer us an example of how the size of nanostructured domains in a semiconductor material can have a drastic effect on the material properties [77,78]. Namely, by tuning the particle size, one modifies the peak emission frequency and the electronic configuration of the material as a whole. As far as the human body is concerned, immunological and toxicological studies have shown that the host response may drastically vary depending on the particle size of an active substance [79,80,81]. In the context of diagnostics and therapeutics, such a wide range of attainable properties, which are tunable depending on the domain size, offers opportunities for a variety of bioengineering applications [82]. In the case of biomaterials, nanoparticulate materials appear to strongly influence the host response at both cellular and tissue levels, which makes nanotechnology particularly attractive for dental implants [83]. Fabrication of nanostructures has been intensively explored in attempt to develop the right combinations of chemistry, microstructure and topography to increase the surface osseoconductivity and at the same time improve the durability and resistance to failure, which are the main limitations of the actual dental implants and coatings [84]. Processes such as sol-gel deposition [85], pulsed laser deposition [86], sputtering coating techniques [87,88], ion beam assisted deposition [61,89,90,91], and electrophoretic deposition [62] are a few examples of nanotechnology-based approaches that have been used to develop bioceramic thin-film coatings for implant surfaces. The main objective of these newer technologies is to reduce the thickness and the particle size of the coating layer and thereby increase its specific surface area and reactivity, thus improving the interaction with the surrounding living tissues. As an alternative to the abovementioned techniques, discrete crystalline depositions [92,93,94] and the combination of resorbable bioceramics with acid etching have been developed with the ability to incorporate calcium and phosphate ions into implant surface layers at nanometer scale [95]. The emerging technology associated with nanostructured bioceramic coatings is primarily related to manipulating surface topographies and chemistries to increase surface osseoconductivity [95]. This technology is under active basic and clinical investigation with the aim to optimize the surface chemistry and texturing at the nanoscale for the best possible clinical outcome [95].

Nanosciences have also recently promoted emerging concepts in oral microbial ecology, which may soon redefine our understanding of biofilm formation and treatment. Recent analyses with ribosomal RNA-based technologies have revealed the diversity of bacterial populations within dental biofilms, and have highlighted their important contributions to oral health and disease [96]. It has been recently suggested that most of the conditions under which oral biofilms develop are tightly linked to the overall health and biology of the host [96]. Advances in molecular techniques have led to a greater appreciation of the diversity of human microbiota, the extent of interactions with the human host, and how that relates to inter-individual variation [97]. Recent studies by Hojo *et al.* demonstrated that there may be no difference in the type and proportion of the present cariogenic bacteria between patients prone to caries and the caries-free ones, thus shifting the focus towards the synergetic interaction of multiple components that reside in the oral cavity [97]. As a consequence, intra-oral events such as plaque formation or the cariogenic challenge may no longer be thought of as a generic process, but rather as a highly individualized process, which has ramifications for the treatment of the diseases it causes [97].

The field of diagnostics of oral diseases is also subject of rapid evolution. Proteomic analyses by mass spectrometry with their ability to identify proteins at ultralow concentration levels have a chance of drastically improving the diagnostic sensitivity and efficiency [98]. Polarization-sensitive optical coherence tomography, polarized Raman spectroscopy, and near-infrared light spectroscopy also hold great promise in the early detection of caries and imaging of developmental abnormalities [99,100,101,102]. Saliva is now recognized as an excellent diagnostic medium for the detection of malignant tumors that are either within or are remote from the oral cavity [103]. The role of salivary glands in controlling the equilibrium between de- and re-mineralization in a cariogenic environment has been well documented [104]. Containing biomarkers for various diseases, the identification of which is currently under investigation, saliva holds great promises for early detection of disease and/or monitoring therapeutic outcomes through a non-invasive approach [105]. Other oral components, such as gingival crevicular fluid, epithelial cells, breath and dental plaque also have diagnostic potential [106]. Since it is a rule of the thumb that cancer leads to alkanes in breath, diabetes leads to acetone, kidney disease leads to ammonia and asthma leads to acetic acid, one may expect that propensity to exhibit specific dental condition based on the dominant chemical in breath to result. Acknowledging oral fluids and tissues as natural tools for health surveillance naturally places dental phenomena in a wider context of the biological integrity of the organism [13]. The future of dentistry will thus undoubtedly witness routine and mechanistic restorations ceding place to a more holistic clinical practice where each particular case is analysed in the context of the organism as a whole.

Finally, in parallel with the strong shift in the field of chemistry away from the traditional reference to strong, chemical bonding effects to the control of weak physicochemical interactions [107] (that has given rise to the prosperous practical framework of self-assembly and soft/wet chemistry [108]), a similar shift away from the mechanically interfering reparative methods towards soft re-mineralization techniques can be said to present one of the most promising streams in the modern dental science. In enamel, the aim appears to be unraveling the ways to mimic Nature’s own nanotechnological mechanism by which the cooperative interaction between the nanoscale self-assemblies of amelogenin and the uniaxially oriented apatite crystals proceeds [3,26]. Dentin, on the other hand, is linked to far more challenging scenarios. Recent studies in our groups now offer clues about novel acellular approaches for tissue-engineering-based repair and augmentation of dentin using partially mineralized collagen scaffolds and mineralization systems with promising results [7]. However, there still appears to be a long and tortuous path to make a step from promising results to the actual transition of a dental tissue engineering methodology from the lab bench to the clinical setting. Despite the seemingly slow development of the dental field, we should keep in mind that scientific fields develop in waves. Computer science has rapidly expanded in the previous two decades or so, whereas the theoretical physics set the quantum mechanical fundaments for its slow subsequent development in only a few decades at the turn of the 20th Century. Let us hope that one such big breaking wave is on the horizon for the world of dental science. In our opinion, to surf on that promising wave, learning the art and know-how offered by modern nanotechnologies will be a must.

## 5. Conclusion

By shifting the main interests in the area of dental research towards modern and innovative approaches that fall into the domain of soft chemical techniques, numerous beneficial consequences might be expected to arise. In this paper we presented some of the developments that might shift the major contemporary interest in dental sciences away from mechanistic approaches for tooth repair to a more profound understanding of teeth restoration and disease-prevention mechanisms. The current cross-disciplinary inclinations of dental research groups to the nanotechnological field, altogether with the major investments aimed towards developing the methods to grow tooth structures artificially by soft chemical approaches and find the mechanisms for remineralization of dental defects at various scales *in vivo*, certainly presents a promising trend. But to be successful in this, a different mindset from the one that dictated dental sciences for a long time may be required.

Long time ago it was said that “the heart of fools is in their mouth, but the mouth of the wise is in their heart” (Sirach 21:26). It is our duty now to believe that dental sciences will not be led astray towards the way of the fools, but will equally well cultivate the way of the wise.

## References

[1] Uskoković V. (2007). Nanotechnologies: What we do not know. Technol. Soc..

[2] Uskoković V. (2008). Nanomaterials and nanotechnologies: Approaching the crest of this big wave. Curr. Nanosci..

[3] Saunders S.A. (2009). Current practicality of nanotechnology in dentistry. Part 1: Focus on nanocomposite restoratives and biomimetics. Clinic. Cosm. Invest. Dent..

[4] Uskoković V. (2009). On the light doves and learning on mistakes. Axiomathes.

[5] Uskoković V. (2009). On science of metaphors and the nature of systemic reasoning. World Futures.

[6] Sano H. (2006). Microtensile testing, nanoleakage, and biodegradation of resin-dentin bonds. J. Dent. Res..

[7] Bertassoni L.E., Habelitz S., Kinney J.H., Marshall S.J., Marshall G.W. (2009). Biomechanical perspective on the remineralization of dentin. Caries Res..

[8] Hansma P., Yu H., Schultz D., Rodriguez A., Yurtsev E.A., Orr J., Tang S., Miller J., Wallace J., Zok F., Li C., Souza R., Proctor A., Brimer D., Nogues-Solan X., Mellbovsky L., Pena M.J., Diez-Ferrer O., Mathews P., Randall C., Kuo A., Chen C., Peters M., Kohn D., Buckley J., Li X., Pruitt L., Diez-Perez A., Alliston T., Weaver V., Lotz J. (2009). The tissue diagnostic instrument. Rev. Sci. Instrum..

[9] Kinney J.H., Habelitz S., Marshall S.J., Marshall G.W. (2003). The importance of intrafibrillar mineralization of collagen on the mechanical properties of dentin. J. Dent. Res..

[10] Hansma P., Turner P., Drake B., Yurtsev E., Proctor A., Mathews P., Lulejian J., Randall C., Adams J., Jungmann R., Garza-de-Leon F., Fantner G., Mkrtchyan H., Pontin M., Weaver A., Brown M.B., Sahar N., Rossello R., Kohn D. (2008). The bone diagnostic instrument II: Indentation distance increase. Rev. Sci. Instrum..

[11] Bertolami C.J. (2002). The role and importance of research and scholarship in dental education and practice. Dent. Educ..

[12] McCoy R.B. (1996). Majestic mediocrity. J. Oper. Dent..

[13] DePaola D.P. (2008). The revitalization of U.S. dental education. J. Dent. Educ..

[14] Iacopino A.M. (2007). The influence of "new science" on dental education: Current concepts, trends, and models for the future. J. Dent. Educ..

[15] Petrini C, Vecchia P. (2002/2003). International statements and definitions of the precautionary principle. IEEE Tech. Soc. Mag..

[16] Reynolds G.H. (2001). Environmental regulation of nanotechnology: Some preliminary observations. Env. Law Rep..

[17] Mnyusiwalla A., Daar A.S., Singer P.A. (2003). “Mind the gap”: Science and ethics in nanotechnology. Nanotechnology.

[18] Smith C.E. (1998). Cellular and chemical events during enamel maturation. Crit. Rev. Oral Biol. Med..

[19] Habelitz S., Marshall S.J., Marshall G.W., Balooch M. (2001). Mechanical properties of human dental enamel on the nanometer scale. Arch. Oral. Biol..

[20] Moradian-Oldak J. (2001). Amelogenins: Assembly, processing and control of crystal morphology. Matrix Biol..

[21] He X., Li W., Habelitz S. (2008). The cooperative self-assembly of 25 and 23 kDa amelogenins. J. Struct. Biol..

[22] Margolis H.C., Beniash E., Fowler C.E. (2006). Role of macromolecular assembly of enamel matrix proteins in enamel formation. J. Dent. Res..

[23] Petta V., Moradian-Oldak J., Yannopoulos S.N., Bouropoulos N. (2006). Dynamic light scattering study of an amelogenin gel-like matrix *in vitro*. Eur. J. Oral. Sci..

[24] Uskoković V., Castiglione Z., Cubas P., Zhu L., Li W., Habelitz S. (2010). Zeta-potential and particle size analysis of recombinant human amelogenins. J. Dent. Res..

[25] Wiedemann-Bidlack F.B., Beniash E., Yamakoshi Y., Simmer J.P., Margolis H.C. (2007). pH triggered self-assembly of native and recombinant amelogenins under physiological pH and temperature *in vitro*. J. Struct. Biol..

[26] Uskoković V., Kim M.K., Li W., Habelitz S. (2008). Enzymatic processing of amelogenin during continuous crystallization of apatite. J. Mater. Res..

[27] Zheng H., Smith R.K., Jun Y.W., Kisielowski C., Dahmen U., Alivisatos A.P. (2009). Observation of single colloidal platinum nanocrystal growth trajectories. Science.

[28] Scaramuzzo F.A., Salvati R., Paci B., Generosi A., Rossi-Albertini V., Latini A., Barteri M. (2009). Nanoscale *in situ* morphological study of proteins immobilized on gold thin films. J. Phys. Chem. B.

[29] Kirkham J., Brookes S.J., Shore R.C., Wood S.R., Smith D.A., Zhang J., Chen H., Robinson C. (2002). Physico-chemical properties of crystal surfaces in matrix-mineral interactions during mammalian biomineralisation. Curr. Opin. Coll. Inter. Sci..

[30] He L.H., Swain M.V. (2008). Understanding the mechanical behaviour of human enamel from its structural and compositional characteristics. J. Mech. Behav. Biomed. Mater..

[31] Stupp S.I., Braun P.V. (1997). Molecular manipulation of microstructures: Biomaterials, ceramics, and semiconductors. Science.

[32] Mann S. (2001). Biomineralization: Principles and Concepts in Bioinorganic Materials Chemistry.

[33] White S.N., Luo W., Paine M.L., Fong H., Sarikaya M., Snead M.L. (2001). Biological organization of hydroxyapatite crystallites into a fibrous continuum toughens and controls anisotropy in human enamel. J. Dent. Res..

[34] Wahl D.A., Czernuszka J.T. (2006). Collagen-hydroxyapatite composites for hard tissue repair. Eur. Cell. Mater..

[35] Sachlos E., Czernuszka J.T. (2003). Making tissue engineering scaffolds work review: The application of solid freeform fabrication technology to the production of tissue engineering scaffolds. Eur. Cell. Mater..

[36] Dibdin G.H. (1993). The water in human dental enamel and its diffusional exchange measured by clearance of tritiated water from enamel slabs of varying thickness. Caries Res..

[37] Weiner S., Traub W. (1992). Bone structure: From angstroms to microns. FASEB J..

[38] Katz E.P., Li S.T. (1973). Structure and function of bone collagen fibrils. J. Mol. Biol..

[39] Katz E.P., Wachtel E., Yamauchi M., Mechanic G.L. (1989). The structure of mineralized collagen fibrils. Connect. Tissue Res..

[40] Landis W.J. (1996). Mineral characterization in calcifying tissues: Atomic, molecular and macromolecular perspectives. Connect. Tissue Res..

[41] Landis W.J., Song M.J., Leith A., McEwen L., Mc-Ewen B.F. (1993). Mineral and organic matrix interaction in normally calcifying tendon visualized in three dimensions by high-voltage electron microscopic tomography and graphic image reconstruction. J. Struct. Biol..

[42] Balooch M., Habelitz S., Kinney J.H., Marshall S.J., Marshall G.W. (2008). Mechanical properties of mineralized collagen fibrils as influenced by demineralization. J. Struct. Biol..

[43] Jager I., Fratzl P. (2000). Mineralized collagen fibrils: A mechanical model with a staggered arrangement of mineral particles. Biophys. J..

[44] Kinney J.H., Pople J.A., Driessen C.H., Breunig T.M., Marshall G.W., Marshall S.J. (2001). Intranfibrillar mineral may be absent in dentinogenesis imperfecta type II (DI-II). J. Dent. Res..

[45] Nalla R.K., Porter A.E., Daraio C., Minor A.M., Radmilovic V., Stach E.A., Tomsia A.P., Ritchie R.O. (2005). Ultrastructural examination of dentin using focused ion beam cross-sectioning and transmission electron microscopy. Micron.

[46] Boyde A. (1974). Transmission electron microscopy of ion beam thinned dentine. Cell. Tissue Res..

[47] Lowenstam H.A., Weiner S. (1989). On Biomineralization.

[48] Nakabayashi N., Kojima K., Masuhara E. (1982). The promotion of adhesion by the infiltration of monomers into tooth substrates. J. Biomed. Mater. Res..

[49] Sano H., Yoshiyama M., Ebisu S., Burrow M.F., Takatsu T., Ciucchi B., Carvalho R., Pashley D.H. (1995). Comparative SEM and TEM observations of nanoleakage within the hybrid layer. Oper. Dent..

[50] Sano H., Takatsu T., Ciucchi B., Horner J.A., Matthews W.G., Pashley D.H. (1995). Nano-leakage: Leakage within the hybrid layer. Oper. Dent..

[51] Tay F.R., Hashimoto M., Pashley D.H., Peters M.C., Lai S.C., Yiu C.K., Cheong C. (2003). Aging affects two modes of nanoleakage expression in bonded dentin. J. Dent. Res..

[52] Hashimoto M., De Munck J., Ito S., Sano H., Kaga M., Oguchi H., Van Meerbeek B., Pashley D.H. (2004). *In vitro* effect of nanolekage expression on resin-dentin bond strengths analysed by microtensile bond test, SEM/EDX and TEM. Biomaterials.

[53] Lees S. (1987). Considerations regarding the structure of the mammalian mineralized osteoid from viewpoint of the generalized packing model. Connect. Tissue Res..

[54] Fawzy A.S., Farghaly A.M. (2009). Probing nano-scale adhesion force between AFM and acid demineralized intertubular dentin: Moist *versus* dry dentin. J. Dent..

[55] Hoffler C.E., Guo X.E., Zysset P.K., Goldstein S.A. (2005). An application of nanoindentation technique to measure bone tissue lamellae properties. J. Biomech. Eng..

[56] Bembey A.K., Bushby A.J., Boyde A., Ferguson V.L., Oyen M.L. (2006). Hydration effects on the micro-mechanical properties of bone. J. Mat. Res..

[57] Kishen A., Vedantam S. (2007). Hydromechanics in dentine: Role of dentinal tubules and hydrostatic pressure on mechanical stress-strain distribution. Dent. Mat..

[58] Cooper A., Allen G. (1999). Thermodynamics of protein folding and stability. Protein: A Comprehensive Treatise.

[59] Marshall G.W., Marshall S.J., Kinney J.H., Balooch M. (1997). The dentin substrate: Structure and properties related to bonding. J. Dent..

[60] Hamvas B. (2002). Scientia Sacra.

[61] Qin C., D’Souza R., Feng J.Q. (2007). Dentin matrix protein 1(DMP1): New and important roles for biomineralization and phosphate homeostasis. J. Dent. Res..

[62] Butler W.T., Brunn J.C., Qin C. (2003). Dentin extracellular matrix (ECM) proteins: Comparison to bone ECM and contribution to dynamics of dentinogenesis. Connect. Tissue Res..

[63] Deshpande A.S., Beniash E. (2008). Bio-inspired synthesis of mineralized collagen fibrils. Cryst. Growth Des..

[64] Bartlett J.D., Ganss B., Goldber M., Moradian-Oldak J., Paine M.L., Snead M.L., Wen X., White S.N., Zhou Y.L. (2006). Protein-protein interactions of the developing enamel matrix. Curr. Top. Dev. Biol..

[65] Gajjeraman S., He G., Narayanan K., George A. (2008). Biological assemblies provide novel templates for the synthesis of hierarchical structures and facilitate cell adhesion. Adv. Funct. Mater..

[66] Ahmad G., Dickerson M.B., Church B.C., Cai Y., Jones S.E., Naik R.R., King J.S., Summers C.J., Kröger N., Sandhage K.H. (2006). Rapid, room-temperature formation of crystalline calcium molybdate phosphor microparticles via peptide-induced precipitation. Adv. Mater..

[67] Dickerson M.B., Naik R.N., Stone M.O., Cai Y., Sandhage K.H. (2004). Identification of peptides that promote the rapid precipitation of germania nanoparticle networks via use of a peptide display library. Chem. Commun..

[68] Marsh P.D., Percival R.S. (2006). The oral microflora–friend or foe? Can we decide?. Int. Dent. J..

[69] Ho S.P., Marshall S.J., Ryder M.I., Marshall G.W. (2007). The tooth attachment mechanisms defined by structure, chemical composition and mechanical properties of collagen fibers in the periodontium. Biomaterials.

[70] Fantner G.E., Hassenkam T., Kindt J.H., Weaver J.C., Birkedal H., Pechenik L., Cutroni J.A., Cidade G.A., Stucky G.D., Morse D.E., Hansma P.K. (2005). Sacrificial bonds and hidden length dissipate energy as mineralized fibrils separate during bone fracture. Nat. Mater..

[71] Hansma P.K., Fantner G.E., Kindt J.H., Thurner P.J., Schitter G., Turner P.J., Udwin S.F., Finch M.M. (2005). Sacrificial bonds in the interfibrillar matrix of bone. J. Musc. Neur. Inter..

[72] Adams J., Fantner G.E., Fisher L.W., Hansma P.K. (2008). Molecular energy dissipation in nanoscale networks of dentin matrix protein 1 is strongly dependent on ion valence. Nanotechnology.

[73] Xu H.H., Moreau J.L., Sun L., Chow L.C. (2008). Strength and fluoride release characteristics of a calcium fluoride based dental nanocomposite. Biomaterials.

[74] Rahiotis C., Vougiouklakis G. (2007). Effect of a CPP-ACP agent on the demineralization and remineralization of dentine *in vitro*. J. Dent..

[75] Gower L.B. (2008). Biomimetic model systems for investigating the amorphous precursor pathway and its role in biomineralization. Chem. Rev..

[76] Gebeshuber I.C., Gruber P., Drack M. (2009). A gaze into the crystal ball—biomimetics in the year 2059. Proc. IMechE. C: J. Mech. Eng. Sci..

[77] Delehanty J.B., Boeneman K., Bradburne C.E., Robertson K., Medintz I.L. (2009). Quantum dots: A powerful tool for understanding the intricacies of nanoparticle-mediated drug delivery. Expert Opin. Drug Deliv..

[78] Pons T., Mattoussi H. (2009). Investigating biological processes at the single molecule level using luminescent quantum dots. Ann. Biomed. Eng..

[79] Singh N., Manshian B., Jenkins G.J.S., Griffiths S.M., Williams P.M., Maffeis T.G.G., Wright C.J., Doak S.H. (2009). NanoGenotoxicology: The DNA damaging potential of engineered nanomaterials. Biomaterials.

[80] Service F. (2003). Nanomaterials show signs of toxicity. Science.

[81] Limbach L.K., Li Y., Grass R.N., Brunner T.J., Hintermann M.A., Muller M., Gunther D., Stark W.J. (2005). Oxide nanoparticle uptake in human lung fibroblasts: Effect of particle size, agglomeration and diffusion at low concentration. Env. Sci. Tech..

[82] Riehemann K., Schneider S.W., Luger T.A., Godin B., Ferrari M., Fuchs H. (2009). Nanomedicine–challenge and perspectives. Angew. Chem. Int. Ed. Engl..

[83] Coelho P.G., Lemons J.E. Bio nano materials. Proceedings of the Bio Nano Conference & Trade Show.

[84] Mendonca G., Mendonca D.B., Aragao F.J., Cooper L.F. (2008). Advancing dental implant surface technology—from micron- to nanotopography. Biomaterials.

[85] Lacefield W.R. (1998). Current status of ceramic coatings for dental implants. Implant Dent..

[86] Kim H., Camata R.P., Vohra Y.K., Lacefield W.R. (2005). Control of phase composition in hydroxyapatite/tetracalcium phosphate biphasic thin coatings for biomedical applications. J. Mater. Sci. Mater. Med..

[87] Vercaigne S., Wolke J.G., Naert I., Jansen J.A. (2000). A mechanical evaluation of TiO_2_-gritblasted and Ca-P magnetron sputter coated implants placed into the trabecular bone of the goat: Part 1. Clin. Oral Implants Res..

[88] Vercaigne S., Wolke J.G., Naert I., Jansen J.A. (2000). A histological evaluation of TiO2-gritblasted and Ca-P magnetron sputter coated implants placed into the trabecular bone of the goat: Part 2. Clin. Oral Implants Res..

[89] Yang Y., Kim K.H., Ong J.L. (2005). A review on calcium phosphate coatings produced using a sputtering process-An alternative to plasma spraying. Biomaterials.

[90] Park Y.S., Yi K.Y., Lee I.S., Han C.H., Jung Y.C. (2005). The effects of ion beam-assisted deposition of hydroxyapatite on the grit-blasted surface of endosseous implants in rabbit tibiae. Int. J. Oral Maxil. Impl..

[91] Ong J.L., Carnes D.L., Bessho K. (2004). Evaluation of titanium plasma-sprayed and plasma sprayed hydroxyapatite implants *in vivo*. Biomaterials.

[92] Mendes V.C., Moineddin R., Davies J.E. (2007). The effect of discrete calcium phosphate nanocrystals on bone-bonding to titanium surfaces. Biomaterials.

[93] Davies J.E. (2007). Bone bonding at natural and biomaterial surfaces. Biomaterials.

[94] Orsini G., Piatelli M., Scarano A., Petrone G., Kenealy J., PIatelli A., Caputi S. (2006). Histologic and ultrastructural analysis of regenerated bone in maxillary sinus augmentation using a porcine bone-derived biomaterial. J. Periodontol..

[95] Coelho P.G., Granjeiro J.M., Romanos G.E., Suzuki M., Silva N.R., Cardaropoli G., Thompson V.P., Lemons J.E. (2009). Basic research methods and current trends of dental implant surfaces. J. Biomed. Mater. Res. B Appl. Biomater..

[96] Filoche S., Wong L., Sissons C.H. (2010). Oral Biofils: Emerging concepts in microbial ecology. J. Dent. Res..

[97] Hojo K., Nagaoka S., Ohshima T., Maeda N. (2009). Bacterial interactions in dental biofilm development. J. Dent. Res..

[98] Hu S., Loo A.J., Wong D.T. (2007). Human saliva proteome analysis and disease biomarker discovery. Expert. Rev. Proteomics.

[99] Choo-Smith L.-P., Dong C.C., Cleghorn B., Hewko M. (2008). Shedding new light on early caries detection. J. Can. Dent. Assoc..

[100] Choo-Smith L.-P., Ko A.C.-T., Hewko M.D., Dong C.C.S., Cleghorn B.M., Sowa M.G., Reichmann P., Fried D. (2006). Characterization of early dental caries by polarized Raman spectroscopy. Lasers in Dentistry XII.

[101] Hirasuna K., Fried D., Darling C.L. (2008). Near-infrared imaging of developmental defects in dental enamel. J. Biomed. Opt..

[102] Fried D., Featherstone J.D., Darling C.L., Jones R.S., Ngaotheppitak P., Buhler C.M. (2005). Early caries imaging and monitoring with near-infrared light. Dent. Clin. North Am..

[103] Bigler L.R., Streckfus C.F., Dubinsky W.P. (2009). Salivary biomarkers for the detection of malignant tumors that are remote from the oral cavity. Clin. Lab. Med..

[104] Edgar W.M., Higham S.M. (1995). Role of saliva in caries models. Adv. Dent. Res..

[105] Lee Y.H., Wong D.T. (2009). Saliva: An emerging biofluid for early detection of diseases. Am. J. Dent..

[106] Ligtenberg A.J., de Soet J.J., Veerman E.C., Amerongen A.V. (2007). Oral diseases: From detection to diagnostics. Ann. N. Y. Acad. Sci..

[107] Uskoković V. (2007). Theoretical and practical aspects of colloid science and self-assembly phenomena revisited. Rev. Chem. Eng..

[108] Tirrell M.V., Katz A. (2005). Self-assembly in materials synthesis. MRS Bull..

